# Introduction time of highly allergenic foods to the infant diet in a UK cohort and association with a family history of allergy

**DOI:** 10.1038/s41430-025-01617-x

**Published:** 2025-04-30

**Authors:** Suzannah Helps, Gillian Mancz, Taraneh Dean

**Affiliations:** 1 School of Health and Care Professions, University of Portsmouth, Portsmouth, UK; 2 School of Health Sciences, Southampton University, Southampton, UK; 3 London South Bank University, London, UK

**Keywords:** Risk factors, Nutrition, Epidemiology, Lifestyle modification

## Abstract

**Background/Objective:**

To describe the introduction of highly allergenic foods in a UK population sample, and to determine whether the introduction of highly allergenic foods differed in infants with family history of allergy.

**Subjects/Methods:**

A population birth cohort study recruited eligible pregnant women while they were attending an antenatal ultrasound clinic appointment at a UK city hospital. Parent-reported family history of allergy and infant diet were collected through structured interviews at recruitment and postal questionnaires. Parents reported on their infants’ diet and introduction of highly allergenic foods at around 6 months (*n* = 216) and around 12 months (*n* = 193), and infant diet around 24 months of age (*n* = 139).

**Results:**

Most highly allergenic foods were introduced to infants at around 6–9 months. However, nut and egg were introduced much later, and 21% of children had not been exposed to egg and 35% of infants had not been exposed to nuts by 12 months. Family history of allergy did not predict late introduction of any of the highly allergenic foods but infants with a family history of allergy were more likely to have diets that avoided foods due to allergy (most commonly dairy, soya, egg and nuts).

**Conclusions:**

The introduction of egg and nuts was delayed beyond one year of age in a large proportion of infants, and infants with a family history of allergy were more likely to have diets that avoided foods due to allergy. These could be modifiable risk factors for allergy development.

Allergic disease is highly prevalent in the UK and worldwide [1]. Observational studies that reported an association between allergy and early exposure to allergenic foods initially led to recommendations to introduce allergenic foods later into children’s diets as a method of preventing food allergies. However more recent, well controlled RCTs have failed to support food avoidance as a method of allergy prevention and these studies, including Learning About Peanut (LEAP [2]) and Early Introduction of Food to Avoid Intolerance (EAT [3]) have instead shown that early introduction of allergenic foods is associated with lower incidence of food allergies. Simons et al. [4] demonstrated that in a population cohort study, children who were introduced to peanut after 1 year of age were more likely to have a peanut allergy at 3 years of age than children who were introduced to peanut earlier.

In 2008, global allergy prevention guidelines were updated to remove the recommendation to delay the introduction of allergenic foods [5]. In 2017, the National Institute of Allergy and Infectious Disease (NIAID) recommend that high-risk children should be introduced to peanut-containing foods as early as 4–6 months of age [6], and the Scientific Advisory Committee on Nutrition (SACN) report in the UK suggests that the introduction of peanut and egg should start along with other solids [7].

There is limited evidence of the extent to which these updated recommendations have been accepted by the UK population. A recent study showed that they are not being followed in Saudi Arabia, where highly allergenic foods such as peanut and fish were typically not introduced until infants were older than one year [8]. However, in Sweden, since national guidelines were changed to recommend the introduction of allergenic foods in the first year of life, infants have been introduced to allergenic foods earlier and consumed these foods more frequently [9].

Furthermore, there has been limited research investigating whether infants at higher risk of allergic disease (those with a first-degree relative with allergy) are introduced to foods, including highly allergenic foods in a different manner. Van Odijk et al. [10] reported that families with a history of allergic disease did not differ in the timing of foods, including allergenic foods, however Schoetzau et al. [11] reported that mothers with a family risk of eczema delayed introducing solid foods compared to mothers without a family history or eczema, and Venter et al. [12] reported that mothers with a family history of allergy were more likely to breastfeed exclusively for longer, and to avoid the introduction of peanuts for longer than women without a family history of allergy. Grimshaw [13, 14] reported that up until one year, the diet of children with and without food allergies were similar, but by 2 years of age, the two groups had significantly different diets and the non-allergic children were more likely to be eating a diet richer in fruit, vegetables and home-prepared foods. Different diets or delayed introduction of allergenic foods in high-risk infants might affect the development of allergies in infants and could be a modifiable risk factor for allergy development [15, 16].

This paper describes the introduction of highly allergenic foods in a population sample at ~6, 12 and 24 months of age. It also examines these factors in high-risk infants with family history of allergy.

The study aimed to investigate:

- The time and type of foods introduced into infants’ diets in a population cohort
- The association between family history of allergy and time and type of foods introduced to infants’ diet

## Methods

### Recruitment and eligibility

Participants were part of a longitudinal population-based cohort study, the Portsmouth Birth Cohort Project in Portsmouth, UK. Pregnant women were screened to check their eligibility for the registry whilst attending an antenatal ultrasound clinic appointment at Portsmouth’s main hospital between 21st May 2015 and the 24th July 2017.

Individuals were eligible for the study if they met the following criteria: Pregnant women aged 16 or over, with a home address within the city’s eight postcodes, who had sufficient mental capacity to consent; booked to deliver their baby at the main hospital birthing centre, the midwifery led maternity centre or at home; planning to live in in the city for a year after the birth.

To reduce selection bias, eligible women were approached and screened, rather than using a volunteer approach. Interpreter services were available for non-English speakers, however, no women required this service.

Full ethical approval for the Birth Cohort registry was given by the South Central Berkshire B Research Ethics Committee (REC ref: 15/SC/0008), and all mothers gave informed consent. The Birth Cohort Registry was designed to gather data on a wide range of factors impacting on the health and development of babies, although this study is specifically focusing on the areas of the questionnaire related to allergy and diet.

### Participants

**Fig. 1:**
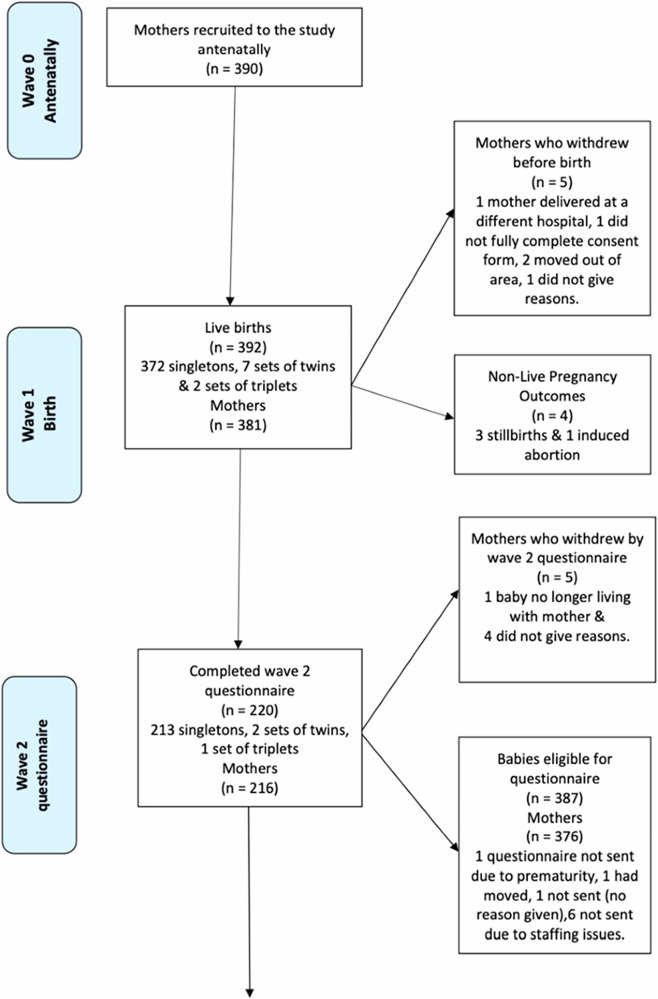

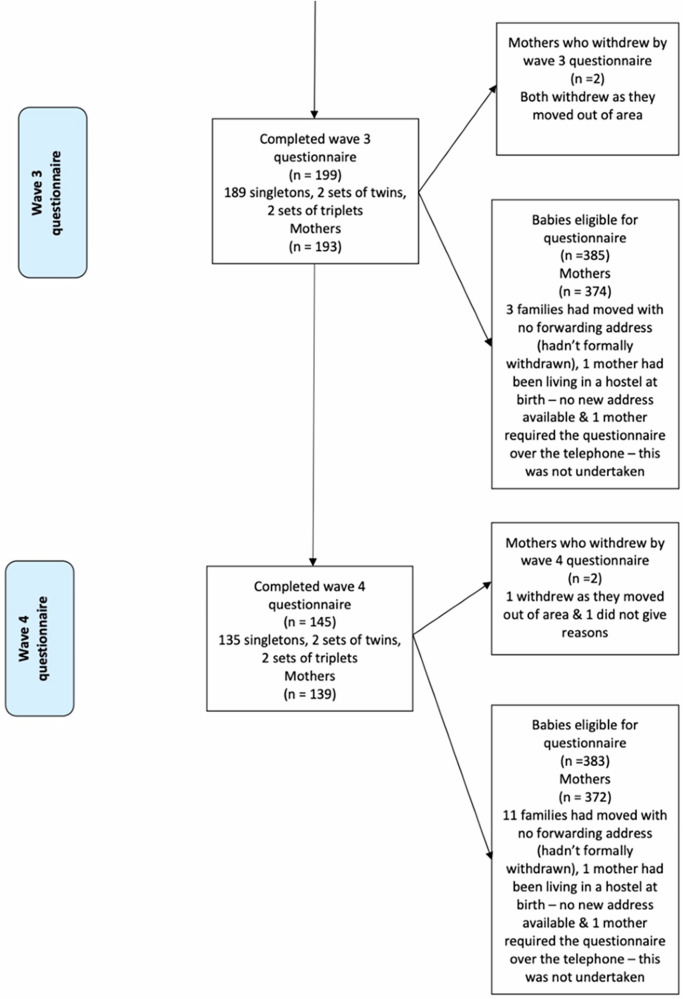
Data collection and participation flow chart.

A total of 390 participants consented to take part in the registry, with one participant joining twice with two different pregnancies (see Fig. 1) for data collection and participation flowchart. This study covers all waves of data collection to date: antenatally (Wave 0), at birth (Wave 1), when the baby was around 6 months (Wave 2), around 12 months (Wave 3), and around 24 months (Wave 4). Only live births were included in the registry for subsequent follow-ups.

Wave 0 data was collected by an interview conducted by a research midwife with the mother in a private area of the antenatal clinic. Wave 1 data were collected by a research midwife from the mother’s medical information. Wave 2, 3 and 4 data were collected using self-completed questionnaires. Paper versions of the questionnaires were sent with self-addressed, stamped envelope, and reminder questionnaires were sent after one month to parents who had not responded.

### Measures

#### Socio-demographic and environmental characteristics

Parental socio-demographic data were collected from the mother antenatally, through the Wave 0 questionnaire, including maternal age, marital status, maternal and paternal education level, maternal and paternal employment status, ethnicity and parity of pregnancy. Wave 1 collected data at birth, including the infant’s sex and date of birth.

#### Family history of allergy

In Wave 0 mother's self-report of family history of allergy was collected (maternal, paternal and sibling history of asthma, hay fever, itchy rash, wheeze, runny nose and food allergies).

#### Infant dietary intake

A parent reported food frequency questionnaire was completed in Waves 2, 3 and 4. This was an amended version of the Southampton Women’s Survey FFQ at 6 months, which has been shown to be a valid measure of energy and nutrient intake in infants [17]. At Wave 2, the questionnaire consisted of a list of 28 foods and 10 drinks; at Waves 3 and 4, the questionnaire consisted of a list of 77 foods and 10 drinks, reflecting the greater range of foods likely to be consumed by an older child. For each food, the frequency of consumption over the previous month of each food and drink was recorded using a multiple-response grid. For each type of food or drink, parents were asked to select how often their infant had eaten or drunk that food in the previous month from the following options on the multiple response grid: Never; 1–3 times; 1,2,3,4,5,6 or 7 times a week; or more than once a day. Variety in the infant’s diet was calculated as the number of different food items consumed over the previous month.

In Waves 2 and 3, parents were also asked to report the age at which their infant was introduced to solid food, by responding to the question ‘When did you first introduce solids into your baby’s diet?’ giving a free text answer of ‘Age in Weeks’. They were also asked the age at which their baby was first introduced to highly allergenic foods by selecting from the options: less than 3 months, 3 to < 6 months, 6 to < 9 months, 9 to <12 months, 12+ months or never, for each of wheat, egg, milk, fish, nuts and sesame. Examples of the kinds of foods that contained each allergen were given for each food type for example, when asked about time of introduction of milk, the following information was provided: Milk, e.g., yoghurt, fromage frais, custard, ice cream, butter, cheese, cow’s milk in foods. For these responses, a single variable was created that considered replies to this question at either wave. If the parent had indicated a different answer in the two Waves, e.g., reported that their infant was introduced to solid food from 20 weeks when asked at Wave 2 but reported 22 weeks when asked at Wave 3, the answer from Wave 2 was used as it was considered less affected by recall bias.

In Waves 2, 3 and 4, parents were asked, ‘Are you avoiding any food in your child’s diet due to allergy?’ and if they responded yes, they were asked to specify which foods using free text.

#### Data analysis

Only questionnaires with complete responses were included in each analysis; those with missing data were excluded from the analysis. SPSS (IBM, version 26) was used to analyse the data. Categorical variables were expressed as frequency and percentage, and the *χ*^2^ test was used to test these relationships. Repeated measures ANOVA was used to test the change in continuous variables (e.g., variety of foods eaten) over time. Binary logistic regressions were used to determine whether family history of allergy was a significant predictor of delayed infants’ introduction to each allergenic food (9 months or later, compared to before 9 months). Maternal and paternal education levels were entered as confounding factors in these models.

## Results

### Demographic characteristics of parents and infants

Demographic characteristics of participants who responded to each wave of data collection are shown in Table 1, as well as the prevalence of allergy among close family members (biological mother, father, or siblings) of the infant. Participants were predominately white British (>80%), which is representative of Portsmouth city (79%). However, respondents to Waves 2–4 were typically highly educated (>70% Higher Education), which is higher than average for Portsmouth’s population (35% Higher Education).

**Table 1:** Parental and infant demographic characteristics of sample.

Parental demographics					
	Total Population	Wave 2 Population	Wave 3 Population	Wave 4 Population	
N	390	216	193	139	
Maternal Age Mean (SD) [Range]	31 years (4.85) [18–43]	33 years (4.38) [19–44]	34 years (4.25) [22–44]	35 years (3.87) [24–45]	
Higher level of Maternal Education*	223 (57%)	151 (70%)	138 (72%)	102 (73%)	*χ*^2^ = 20.7 *p* < 0.05 W2, W3, W4 > Total
Higher level of Paternal Education*	164 (42%)	113 (52%)	99 (51%)	83 (60%)	*ns*
Maternal Ethnicity					
White British	318 (82%)	174 (81%)	161 (83%)	114 (82%)	*ns*
White other	44 (11%)	31 (14%)	26 (13%)	19 (14%)	
Mixed/multiple ethnic groups	6 (2%)	2 (1%)	1 (1%)	1 (1%)	
Asian/Asian British	13 (3%)	6 (3%)	4 (2%)	3 (2%)	
Black/African/Caribbean/Black British	6 (1.5%)	2 (1%)	1 (1%)	1 (1%)	
Other ethnic group	2 (1%)	—	—	—	
Not specified	1 (1%)	1 (1%)	—	1 (1%)	
Infant Demographics					
N	—	220	199	145	
Sex: Male N (%)	—	191 (49%)	113 (51%)	97 (49%)	*ns*
Age (SD) [range] months	—	6 (0.91) [5–10]	13 (1.1) [11–17]	25 (0.99) [23–30]	
Type of Birth					
Natural birth	230 (59%)	132 (60%)	122 (61%)	93 (64%)	*ns*
Caesarean	130 (33%)	65 (30%)	54 (27%)	38 (26%)	
Instrumental	30 (8%)	19 (9%)	18 (9%)	11 (8%)	
Instrumental and caesarean	1 (<1%)	1 (<1%)	1(1%)	1(1%)	
Instrumental and cesarean birth order: firstborn (no siblings)	165 (42%)	108 (50%)	98 (51%)	67 (48%)	*ns*
Variety of food eaten in previous month					
Whole sample	—	11.4 (5.76)	46.6 (9.32)	48.2 (8.77)	F(1, 106) = 1219, *p* < 0.001 W2 < W3 < W4
No family history allergy (NFHA)		10.9 (5.61)	45.6 (10.51)	48.9 (10.9)	NFHA vs FHA *ns*
Family history allergy (FHA)		11.6 (5.83)	46.9 (9.35)	48.4 (8.3)	Group (FHA/NFHA) *X* time *ns*
Consulted GP about any allergic symptoms	—	53 (24%)	79 (40%)	42 (29%)	*χ*^2^ = 7.49 *p* < 0.05
Avoiding any foods due to allergy	—	32 (15%)	32 (16%)	15 (10%)	*ns*
Family history of allergy	(Any close family history)				
Asthma	149 (38%)	69 (32%)	59 (31%)	38 (27%)	*ns*
Hay fever	208 (53%)	114 (53%)	102 (53%)	71 (51%)	*ns*
Itchy rash	117 (30%)	65 (30%)	60 (31%)	40 (29%)	*ns*
Wheeze	140 (36%)	75 (34%)	63 (33%)	34 (24%)	*ns*
Runny nose	183 (47%)	106 (49%)	97 (50%)	66 (47%)	*ns*
Food allergy	110 (28%)	60 (28%)	56 (29%)	41 (29%)	*ns*

Higher level of education refers to having obtained a higher education qualification (normally beyond age 18), which includes bachelor's degrees, as well as degree apprenticeships, and foundation degrees.

Respondents to the questionnaire were older and more likely to have attended higher education compared to non-respondents. However, there was no difference in the percentage of mothers of white British ethnicity for respondents compared to non-respondents

In all waves of data collection, hay fever was the most commonly reported allergy, and more than 50% of infants had at least one close family member with hay fever. Around 28% of infants had a close family member with a food allergy, and 38% of infants had a close family member with asthma.

15% of parents reported that they were avoiding giving their infants certain foods due to allergy at Wave 2, 16% at Wave 3 and 10% at Wave 4 (see Table 1). The most commonly reported foods that were being avoided due to allergy at Wave 2 were: dairy (*n* = 14, yogurt *n* = 1), soya (*n* = 6), egg (*n* = 5), nuts (*n* = 5) and fruits (banana, *n* = 2; strawberries, *n* = 1; orange, *n* = 1). At Wave 3 commonly avoided foods were dairy (*n* = 14, milk and cheese, *n* = 2), egg (*n* = 8), nuts (*n* = 8), soya (*n* = 6) and fruits (banana *n* = 1; blueberries, *n* = 1; watermelon, *n* = 1; cucumber, *n* = 1; and strawberries, *n* = 1). And at Wave 4, the most commonly avoided foods were dairy (*n* = 6), nuts (*n* = 4), egg (*n* = 3), soya (*n* = 3) and fruits (banana, *n* = 2; raspberries, *n* = 1; tomato, n = 2; kiwi, *n* = 1; blueberries, *n* = 1; apricot, *n* = 1).

### Time and type of foods introduced into infants’ diets

The mean age for introduction of solid foods was 22.7 weeks (SD = 3.40, range 6–36 weeks). Most highly allergenic foods were introduced to infants at around 6–9 months. Wheat was introduced earlier than the other allergenic foods: 31% of infants were introduced to wheat before they reached 6 months, and nearly all infants had been introduced to wheat by the time they were 9 months (97%). Nuts were introduced latest with only 38% of infants being introduced to nuts by the time they were 9 months old, and 35% of infants never having exposure to nuts by Wave 3 (around 12 months). Egg and sesame were also introduced to infants later, and 21% of infants had not been exposed to eggs and 16% had not been exposed to sesame by Wave 3 (around 12 months) see Table 2.

**Table 2:** Number of children who were introduced to highly allergenic foods at each age (in months).

	Age (months) N (%)					
	<3	3 to <6	6 to <9	9 to <12	12+	Never
Wheat	2 (1%)	70 (30%)	154 (66%)	4 (2%)	—	2 (1%)
Egg	—	9 (5%)	120 (60%)	22 (11%)	7 (4%)	41 (21%)
Milk	1 (<1%)	34 (16%)	158 (73%)	12 (6%)	4 (2%)	7 (3%)
Fish	—	9 (4%)	134 (63%)	52(25%)	5 (2%)	12 (6%)
Nuts	1 (<1%)	3 (2%)	69 (35%)	41 (21%)	12 (7%)	69 (35%)
Sesame	1 (<1%)	5 (3%)	91 (46%)	52 (26%)	18 (9%)	32 (16%)

Repeated measure ANOVA showed that the variety of foods eaten by infants increased as they aged (see Table 1). Both the increase in variety of foods eaten from Wave 2 to Wave 3, and the increase in Wave 3 to Wave 4, were statistically significant.

### The association between family history of allergy and time, and type of foods introduced to infants’ diet

There was no difference in the age of introduction of solid food between the infants with any family history of any allergy and infants without a family history of these allergies (*t*(135) = 1.47, *p* = 0.144, *ns*).

Table 3 shows the percentage of infants introduced late (9 months or later) to highly allergenic foods by family history of allergy. As shown in Table 4, family history of allergy was not a significant predictor in any of the binary logistic regression models which assessed the likelihood that infants’ introduction to each allergenic food was delayed, Thus, family history of allergy did not make a significant contribution to delayed introduction (9 months or later compared to before 9 month) of any of these allergenic foods.

**Table 3:** Percentage of infants introduced late (9 months or later) to highly allergenic foods by family history of allergy.

	Late introduction of:					
	Wheat	Egg	Milk	Fish	Nuts	Sesame
Asthma	1%	22%	7%	27%	60%	45%
No Asthma	4%	27%	11%	34%	62%	53%
Hay fever	3%	28%	12%	30%	58%	51%
No Hay fever	2%	22%	7%	34%	65%	49%
Rash	4%	31%	14%	27%	60%	44%
No Rash	3%	23%	8%	34%	62%	53%
Food Allergy	5%	24%	8%	37%	69%	47%
No Food Allergy	2%	26%	10%	30%	58%	51%
Wheeze	1%	22%	5%	25%	58%	47%
No Wheeze	3%	27%	12%	35%	63%	52%

**Table 4:** Binary Logistic regressions to assess whether family history of different types of allergy are significant predictors of delayed infants’ introduction to each allergenic food (9 months or later, compared to before 9 months)^a,b^.

	Sig.	Exp(B)	95% C.I. for EXP(B)	Sig.
			Lower	Upper
Delayed introduction of wheat				
Maternal education	0.916	1.120	0.137	9.158
Paternal education	0.190	4.129	0.494	34.472
Family history of asthma	0.797	0.722	0.060	8.658
Family history of hay fever	0.284	2.644	0.447	15.651
Family history of rash	0.403	0.387	0.042	3.583
Family history of food allergy	0.930	1.086	0.170	6.943
Family history of wheeze	0.417	0.371	0.034	4.063
Delayed introduction of egg				
Maternal education	0.734	1.118	0.586	2.133
Paternal education	0.310	0.781	0.485	1.258
Family history of asthma	0.945	1.029	0.460	2.300
Family history of hay fever	0.487	1.254	0.662	2.375
Family history of rash	0.851	1.067	0.543	2.094
Family history of food allergy	0.815	0.921	0.465	1.827
Family history of wheeze	0.633	0.830	0.387	1.782
Delayed introduction of milk				
Maternal education	0.599	0.769	0.289	2.045
Paternal education	0.611	1.215	0.574	2.572
Family history of asthma	0.621	0.726	0.203	2.590
Family history of hay fever	0.141	2.122	0.779	5.782
Family history of rash	0.115	2.153	0.829	5.591
Family history of food allergy	0.442	0.644	0.210	1.978
Family history of wheeze	0.306	0.527	0.155	1.797
Delayed introduction of fish				
Maternal education	**0.014**	**0.450**	**0.238**	**0.852**
Paternal education	0.827	1.055	0.651	1.711
Family history of asthma	0.446	0.713	0.299	1.701
Family history of hay fever	0.732	0.892	0.464	1.714
Family history of rash	0.328	0.705	0.350	1.421
Family history of food allergy	0.155	1.675	0.823	3.412
Family history of wheeze	0.426	0.725	0.329	1.600
Delayed introduction of nut				
Maternal education	0.544	0.819	0.429	1.561
Paternal education	0.757	0.927	0.572	1.502
Family history of asthma	0.958	0.978	0.426	2.245
Family history of hay fever	0.400	0.760	0.402	1.439
Family history of rash	0.713	1.135	0.579	2.227
Family history of food allergy	0.142	1.723	0.834	3.561
Family history of wheeze	0.643	0.833	0.385	1.803
Delayed introduction of sesame				
Maternal education	0.446	0.786	0.424	1.459
Paternal education	0.848	1.048	0.652	1.683
Family history of asthma	0.186	0.577	0.255	1.304
Family history of hay fever	0.271	1.417	0.762	2.634
Family history of rash	0.428	0.768	0.400	1.475
Family history of food allergy	0.971	1.012	0.513	1.999
Family history of wheeze	0.880	0.943	0.442	2.014

^a^Maternal and paternal education were entered as confounding factors in the model.

^b^Total number of observations = 212.

The bold values indicate that *p* < 0.05.

Only one predictor in any of the models was statistically significant. The logistic regression investigating the delayed introduction of fish revealed a significant coefficient for maternal education (*β* = −0.798, *p* = 0.014), which suggests that lower maternal education is associated with a higher likelihood of delayed introduction of fish to infants (after 9 months).

However, as shown in Table 5, at Wave 2, infants with a family history of every type of allergy were more likely to have diets avoiding certain foods due to allergy compared to infants without a family history of that allergy. At Wave 3, infants with a family history of asthma, hay fever, itchy rash or food allergy were more likely to have diets avoiding foods due to allergy and at Wave 4, infants with a family history of asthma or hay fever were more likely to have diets avoiding foods due to allergy, than infants without a family history of these allergies.

**Table 5:** The percentage of infants with diets that avoid food due to allergy, a comparison of infants with and without a family history of allergy.

	Percentage of infants with diets that avoid foods due to allergy		*χ*^2^ (*p* value)
	Family history of asthma	No family history of asthma	
Wave 2	27%	10%	7.68 (*p* = 0.006)
Wave 3	24%	13%	11.05(*p* = 0.001)
Wave 4	23%	6%	9.88 (*p* = 0.002)
	Family history of hay fever	No family history of hay fever	
Wave 2	24%	8%	8.60 (*p* = 0.003)
Wave 3	25%	7%	11.05 (*p* = 0.001)
Wave 4	20%	1%	13.18 (*p* < 0.001)
	Family history of itchy rash	No family history of itchy rash	
Wave 2	25%	12%	4.72 (*p* = 0.030)
Wave 3	25%	7%	11.05 (*p* = 0.001)
Wave 4	13%	10%	0.277 (*p* = 0.599) *ns*
	Family history of wheeze	No family history of wheeze	
Wave 2	27%	11%	9.17 (*p* = 0.002)
Wave 3	23%	14%	2.62 (*p* = 0.106) *ns*
Wave 4	18%	8%	2.55 (*p* = 0.116) *ns*
	Family history of runny nose	No family history of runny nose	
Wave 2	23%	10%	6.75 (*p* = 0.009)
Wave 3	22%	12%	3.15 (*p* = 0.076) *ns*
Wave 4	12%	9%	0.412 (*p* = 0.521) *ns*
	Family history of food allergy	No family history of food allergy	
Wave 2	30%	11%	11.18 (*p* = 0.001)
Wave 3	32%	11%	12.04 (*p* = 0.001)
Wave 4	10%	10%	0.211(*p* = 0.646) *ns*

Repeated measures ANOVA showed that there was no significant difference in the variety of foods eaten by the infants who had a family history of allergy compared to infants without a family history of allergy, and there was also not a significant Group X time interaction (see Table 1).

## Discussion

This study aimed to investigate the time and type of foods introduced into infants’ diets in this population cohort and to determine whether this differed in infants with a family history of allergy compared to infants without a family history of allergy.

Although there is no specific guidance for the optimal age of timing of introduction of highly allergenic foods, current guidelines for primary prevention of food allergy does not advise late introduction of allergenic foods [18–20]. And rather that highly allergenic foods are introduced alongside other solids. Research generally reports that early introduction of allergenic foods, particularly nuts and egg, is associated with lower risk of allergic disease [21], and it has been suggested that high-risk children should be introduced to peanut-containing foods as early as 4–6 months of age [6].

In this sample, most highly allergenic foods were introduced to infants at around 6–9 months. However, some allergenic foods were introduced much later, for example a fifth (21%) of children had not been exposed to egg, and over a third of infants (35%) had not been exposed to nuts by 12 months. This suggests that a significant proportion of parents are not following the recommendations to introduce highly allergenic foods alongside other solid foods, and are delaying the introduction of allergenic foods to their infants. Similarly, between 10 and 16% of infants had diets that avoided foods due to allergy at each wave of data collection.

In this sample, parents of the infants, both those with and without a family history of allergy did not appear to be following advice to introduce nuts and eggs alongside other solids, and nuts and eggs were introduced late, with a large proportion of infants not being exposed to either of these foods by one year of age. Delaying the introduction of nuts and egg in the population and the avoidance of highly allergenic foods in high-risk infants might affect the development of allergies in infants [15, 16]. However, these dietary decisions could be a modifiable risk factor for allergy development: in countries, such as Australia, where a national strategy has been implemented to communicate the recommendations to introduce allergenic foods earlier to infants, early introduction to allergenic foods has been well accepted by the population (see for example the Nip Allergies in the Bub strategy [22] and the Early Nuts Study [23]). Similarly, in Sweden, when the guidelines released by the Swedish National Food agency in 2019 were updated to recommend earlier introduction of allergenic foods, in the first year of life, there was an increase in the percentage of parents who introduced their infants to allergenic foods earlier [15]. However, it is not currently clear whether the introduction of these guidelines is associated with reduced allergy prevalence. In Sweden, prevalence of allergy was not reduced in the cohort of infants recruited after guidelines were introduced recommending earlier introduction of allergenic foods, compared to a cohort of children recruited before these guidelines were introduced [15]. Similarly, in Australia, the prevalence of peanut allergy was not statistically different in cohorts recruited before and after guidelines that recommended early peanut introduction [24]. As well-controlled RCTs [25] have shown that early introduction of allergenic food is associated with reduced allergy prevalence, it may be that these guidelines are not being sufficiently adhered to in the population.

From the current study, it is not clear why parents are not following the current guidance to introduce allergenic foods alongside other foods during weaning, or why parents of infants with a family history of allergy choose to avoid certain foods due to allergy (either allergy in the infant or a family history of allergy), future qualitative research would help to understand the factors underpinning these decisions e.g. [26].

Furthermore, in the current study, although infants with a family history of allergy were introduced to allergenic foods at similar times to other infants, they were more likely to have diets that avoided particular foods than infants without a family history of allergy. The foods that were reported to be avoided by infants were most commonly dairy, soya, egg and nuts, but also included a variety of fruits. This is likely to reflect that infants with a family history of allergy are introduced to allergenic foods at a similar time as other infants, but that their parents later decide to avoid these foods in their infants’ diets. Many of the foods being avoided in the infants’ diets are not highly allergenic foods; for example, at each wave of data collection, a number of parents reported avoiding fruits such as banana, which is uncommonly associated with allergy. This may be due to limited and conflicting information available to parents about infant feeding [26, 27].

One of the strengths of the current study was its longitudinal design and stringent recruitment criteria which was verified by research midwives, this ensured that only parents with infants of an appropriate age were able to enter the study and the parents were unlikely to be affected by recall bias in their reports of when they introduced foods to the infants, as the parents were sent the questionnaires to completed when their infants were of an appropriate age. However, limitations of the study include reliance of parental-report measures, for example, questions about the timing of introductions of highly allergenic foods required parents to understand the ingredients in pre-prepared infant foods. Examples of foods that contained each allergen were provided to mitigate this. Parental-reporting also may have introduced bias as parents could have misunderstood questions or interpreted questions in different ways, for example when parents were asked to report whether they were avoiding foods from their infant’s diet due to allergy, some parents may have reported avoidance based on allergy in their infant and others may have reported avoidance based on family history of allergy. Data in Wave 0 was collected using a structured interview with a research midwife, and data in Wave 1 was collected directly from hospital records, therefore, these waves of data collection are more likely to avoid such biases.

The participants involved in the study were also more likely to be highly educated than the typical Portsmouth population. The response rate to the questionnaires ranged from 36% to 55%, and older, more highly educated mothers from the initial cohort were more likely to respond. These findings may not be replicable in younger, less well-educated populations.

In conclusion, in this sample, although most highly allergenic foods were introduced to infants along with other solid foods, many parents delayed the introduction of egg and nuts beyond one year of age. This suggests that a large proportion of the UK population is not following public health nutrition recommendations to introduce these highly allergenic foods alongside other foods to their infants. Furthermore, infants with a family history of allergy were more likely to have diets that avoided foods due to allergy. These behaviours may contribute to the development of allergic disease.

## Data Availability

The data that support the findings of this study are available on request from the corresponding author. The data are not publicly available due to privacy or ethical restrictions.
